# The relationship between neurotrophins and cognitive functions in the context of emotional response to sleep deprivation

**DOI:** 10.1192/j.eurpsy.2024.811

**Published:** 2024-08-27

**Authors:** M. Ditmer, A. Binienda, A. Tarasiuk, A. Gabryelska, P. Białasiewicz, S. Turkiewicz, F. F. Karuga, J. Fichna, M. Sochal

**Affiliations:** ^1^Department of Sleep Medicine and Metabolic Disorders; ^2^Department of Biochemistry, Medical University of Lodz, Lodz, Poland

## Abstract

**Introduction:**

Studies conducted up to date on the subject of deprivation of sleep (DS) primarily focused either on its impact on certain cognitive abilities or mood-enhancing effects in patients with depression. A notable body of evidence suggests that both might be related to alterations in neurotrophin synthesis induced by DS. However, the role of NTs as an interface between DS, mood, and cognitive functions is unclear.

**Objectives:**

The study aimed to investigate associations between cognitive abilities measured by Trail Making Test (TMT) and Stroop Color and Wort Test (ST), serum protein concentrations of brain-derived neurotrophic factor (BDNF), glial cell line-derived neurotrophic factor (GDNF), neurotrophin-3 (NT3), neurotrophin-4 (NT4) as well as the expression of their respective genes after a night of sleep deprivation.

**Methods:**

Each participant (n=76) underwent a 24-hour DS under the control of actigraphy. Venous blood collection, TMT, and ST were carried out in the morning after DS. Mood was evaluated twice, after DS and in the preceding evening; based on the alleviation of depression symptoms participants were divided into respondents (RE; n=47) and non-respondents (NR; n=29). Serum protein concentration was determined using ELISA kits. Gene expression was evaluated by quantitative real-time polymerase chain reaction with gene-specific probes (reference gene: β-actin). Relative expression was calculated using the Livak formula. TMT is a neuropsychological instrument; Part 1 is thought to evaluate mostly attention, whereas Part 2 executive functions. ST is a 2-part test applied in the assessment of response inhibition and complex attention.

**Results:**

In RE, cognitive abilities were not associated with expression levels of any of the studied proteins or mRNA (all p>0.05). In NR, BDNF and GDNF mRNA expressions negatively correlated with TMT Part 1 (p=0.017, p=0.048, respectively); scores obtained in TMT Part 2 bore a similar relation to BDNF, GDNF, and NT4 mRNA (p=0.034, p=0.041, p=0.026, respectively). In this group, expression of all BDNF, GDNF, NT3, NT4 mRNA correlated negatively with both parts of ST (p<0.001, p=0.009, p=0.042, p=0.009 for Part 1; p<0.001, p=0.003, p=0.031, p=0.014 for Part 2, respectively).

**Conclusions:**

Those results suggest that alterations in the synthesis of NTs might be an element of the molecular milieu characterizing different types of DS response. Negative correlations between test scores and NT mRNA expressions could imply that the reduction of the production of NT proforms might protect against the decline of cognitive functions in the aftermath of DS. Projects using a larger battery of tests as well as analyzing immature forms of NTs would be desirable in order to further investigate mechanisms underlying DS response.

**Disclosure of Interest:**

None Declared

